# Long-Term Mortality and Survival in Patients with Acute Heart Failure Assessed by Emergency Medical Services

**DOI:** 10.3390/jcdd12120485

**Published:** 2025-12-10

**Authors:** Enrique Castro-Portillo, Ana Ramos-Rodríguez, Raúl López-Izquierdo, Irene Bermudez-Castellanos, Miguel Á. Castro Villamor, Santiago Otero de la Torre, Francisco T. Martínez Fernández, Irene Sánchez Soberon, Ancor Sanz-García, Francisco Martín-Rodríguez

**Affiliations:** 1 Primary Health Care Unit, Centro de Salud Delicias II, Gerencia Regional de Salud de Castilla y León, 47001 Valladolid, Spain; 2 Emergency Department, Hospital Universitario Rio Hortega, Gerencia Regional de Salud de Castilla y León, 47001 Valladolid, Spain; aramosrodri@saludcastillayleon.es (A.R.-R.);; 3 Faculty of Medicine, Universidad de Valladolid, 47005 Valladolid, Spain; mcastrovi@saludcastillayleon.es (M.Á.C.V.);; 4 Ophthalmology Department, Hospital Universitario Rio Hortega, Gerencia Regional de Salud de Castilla y León, 47001 Valladolid, Spain; 5 Primary Health Care Unit, Centro de Zaratán, Gerencia Regional de Salud de Castilla y León, 47001 Valladolid, Spain; 6 Prehospital Critical Care, Emergency Medical Services, Gerencia Regional de Salud de Castilla y León, 47001 Valladolid, Spain; 7 Technological Innovation Applied to Health Research Group (ITAS Group), Faculty of Health Sciences, University of de Castilla-La Mancha, 45600 Talavera de la Reina, Spain; ancor.sanz@gmail.com; 8 Group of Healthcare Asessment, Instituto de Investigación Sanitaria de Castilla-La Mancha (IDISCAM), 45004 Toledo, Spain; 9 Faculty of Health Sciences, University of Castilla la Mancha, 45600 Talavera de la Reina, Spain

**Keywords:** acute heart failure, prehospital care, long-term mortality

## Abstract

**Background:** Acute heart failure (AHF) is a common reason for emergency care, yet data on its epidemiology and prognosis in the prehospital setting remain limited. This study aims to analyze the characteristics and long-term survival outcomes of patients with AHF managed by emergency medical services (EMSs). **Methods**: A multicenter, prospective, observational study was conducted in adult patients attended by EMSs and transferred to emergency departments (EDs). Collected data included demographics, vital signs, laboratory parameters, chronic obstructive pulmonary disease (COPD) history, comorbidity burden assessed using the Age-adjusted Charlson Comorbidity Index (aCCI), and clinical outcomes. The primary endpoint was 2-year mortality (M2Y). Survival analysis was performed using Cox regression and Kaplan–Meier analysis. **Results**: A total of 417 patients were included. Their median age was 84 years, and 48.2% were women. A total of 92.3% of the patients had an elevated aCCI. Overall, M2Y was 57.6%, rising to 74.4% among COPD patients. aCCI range and elevated plasma potassium and lactate levels were independently associated with reduced survival (HR 2.86, 1.45 and 1.15, respectively). Overall, 50% of all deaths occurred within the first 49 days. **Conclusions**: AHF patients attended by EMSs exhibited high 2-year mortality, likely due to advanced age and comorbidities. High comorbidity burden and abnormal potassium and lactate levels were linked to worse outcomes.

## 1. Introduction

Acute cardiovascular diseases (ACVDs) represent the most frequently encountered group of pathologies in the daily clinical practice of emergency medical services (EMSs). Notably, in 2019, 43% of the incidents attended by mobile emergency units were attributed to cardiovascular disorders [1].

Heart failure (HF) is a clinical syndrome defined by a variety of symptoms and signs frequently caused by structural and/or functional cardiac abnormality resulting in reduced cardiac output or elevated intracavitary pressures [2]. Although the incidence of HF has slightly decreased over time, its overall prevalence continues to rise, driven by advances in its management and increased life expectancy. In European countries, the median incidence rate is 3.2 cases per 1000 person-years, while the median prevalence reaches 17.2 cases per 1000 individuals. Despite advances in treatment, the prognosis of HF remains poor, though some improvement has been seen over recent decades. Mortality rates remain high, with 5-year rates exceeding 50% in population-based studies [3]. Furthermore, its economic burden on health care systems is high. HF-related health care costs in the United States of America, estimated at around 30 billion dollars in 2012, are projected to rise by 127%, reaching approximately 70 billion dollars by 2030 [4]. Its acute presentation, acute heart failure (AHF), is the second most common medical condition assessed by EMSs and generates 5–6% of all pre-hospital alerts [5]. It also represents between 20.5 and 25% of the patients with dyspnea treated by EMSs [6,7].

Despite the high prevalence and severity of AHF, there is little evidence regarding the epidemiology and long-term prognosis of patients managed for AHF in the prehospital setting. The primary aim of this study is to investigate the characteristics and long-term survival outcomes of patients with AHF managed by EMSs.

## 2. Materials and Methods

### 2.1. Design

A prospective, observational, multicenter study was conducted in adult patients with suspected AHF attended by EMSs and subsequently transferred by ambulance to hospital EDs between 1 October 2019 and 30 September 2024. This study followed the STROBE (Strengthening the Reporting of Observational Studies in Epidemiology) guidelines to ensure methodological rigor (see Supplementary Materials Table S1). The research protocol was reviewed and approved by the ethics committees of all participating institutions (references: PI-GR-19-1258 and PI-041-19).

### 2.2. Setting

This study was conducted across three provinces in Spain (Salamanca, Segovia, and Valladolid) encompassing a reference population of approximately 1,012,077 individuals. It involved five Advanced Life Support (ALS) units, forty-eight Basic Life Support (BLS) units, and four EDs, three of which were located in tertiary hospitals and one in a regional hospital. All participating services were coordinated under the Castilla y León Public Health System (SACYL). Patients requiring urgent medical care-initiated contact via the emergency number 1-1-2, through which operators collected geolocation and identification data. A coordinating physician then conducted a brief clinical assessment and dispatched the most suitable response team. BLS teams were staffed by two Emergency Medical Technicians (EMTs), while ALS teams included two EMTs, an Emergency Registered Nurse (ERN), and a physician. Care was delivered following standardized protocols and clinical guidelines, either at the site of the incident or during patient transport.

### 2.3. Participants

This study included adult patients (aged 18 years or older) who received a prehospital diagnosis of AHF and were transferred to an ED by ambulance. Initial clinical assessment was performed on-site by an ALS team. Based on this evaluation, the attending ALS physician determined whether the patient could be safely discharged at the scene (typically in cases suitable for outpatient management) or required hospital transfer via ALS or BLS units. Exclusion criteria comprised patients under 18 years of age, unresolved cardiac arrest, confirmed or suspected pregnancy, terminal illness documented by a specialist, and absence of informed consent.

### 2.4. Outcome

The primary objective of this study was to analyze the survival of patients with AHF over a two-year follow-up period from the index event, defined as the date of initial prehospital emergency medical care. The main outcome measure was all-cause mortality at two years.

### 2.5. Data

During the initial patient assessment, the ALS ERN recorded baseline vital signs on site using a LifePAK^®^ 15 defibrillator-monitor (Physio-Control, Inc., Redmond, DC, USA). Vital signs included blood pressure, heart rate (HR), respiratory rate (RR), oxygen saturation (SpO_2_), fraction of inspired oxygen (FiO_2_), temperature (TT), and Glasgow Coma Scale (GCS). TT was measured specifically with a ThermoScan^®^ PRO 6000 (Welch Allyn, Inc., Skaneateles Falls, NY, USA). Additionally, electrocardiographic data and key epidemiological variables (such as age, sex, institutionalization status, and referral from Primary Care) were documented. A venous blood sample was collected at the same time and analyzed using an Epoc^®^ analyzer (Siemens Healthcare GmbH, Erlangen, Germany) to determine biomarkers including glucose, sodium, potassium, lactate, creatinine, troponins, D-dimer, NT-proBNP and urea levels. Information regarding the type of oxygen therapy and ventilation method employed, when applicable, as well as the initial diagnostic impression, was also recorded. Throughout the two-year follow-up period, investigators at each ED reviewed electronic health records to gather additional hospital data. This included patients’ history of chronic obstructive pulmonary disease (COPD), comorbidities for calculation of the age-adjusted Charlson Comorbidity Index (aCCI; see Supplementary Table S2), hospital admissions, intensive care unit (ICU) stays, and mortality outcomes at one year (M1Y) and two years (M2Y).

### 2.6. Data Analysis

Normality of the variables analyzed was checked using the Kolmogorov–Smirnov and Shapiro–Wilk tests. Descriptive results and the association between variables and outcomes were performed using the Mann–Whitney U test or the chi-square test when appropriate. Medians and interquartile ranges (IQRs) between the 25th–75th percentiles were used to describe quantitative variables, as they did not follow a normal distribution, while absolute values and percentages were used for categorical variables. Based on previous studies [8], a mortality of 55.4% was estimated in a reference population of 1334 inhabitants. Assuming a precision of 4%, an alpha error probability of 5% and a power of 80%, the calculated sample size was at least 594 patients. Finally, a survival analysis was conducted using the Kaplan–Meier method to estimate cumulative survival over time. Additionally, a Cox proportional hazards regression model was used to assess the impact of individual variables on mortality. Initially, a univariate Cox analysis was performed to identify those variables significantly associated with survival. Variables that demonstrated a statistically significant association (*p* < 0.05) in the univariate analysis were subsequently included in a multivariate Cox regression model to determine their independent contribution to mortality risk, controlling for potential confounders.

## 3. Results

### 3.1. Sample Characteristics

**Figure 1:**
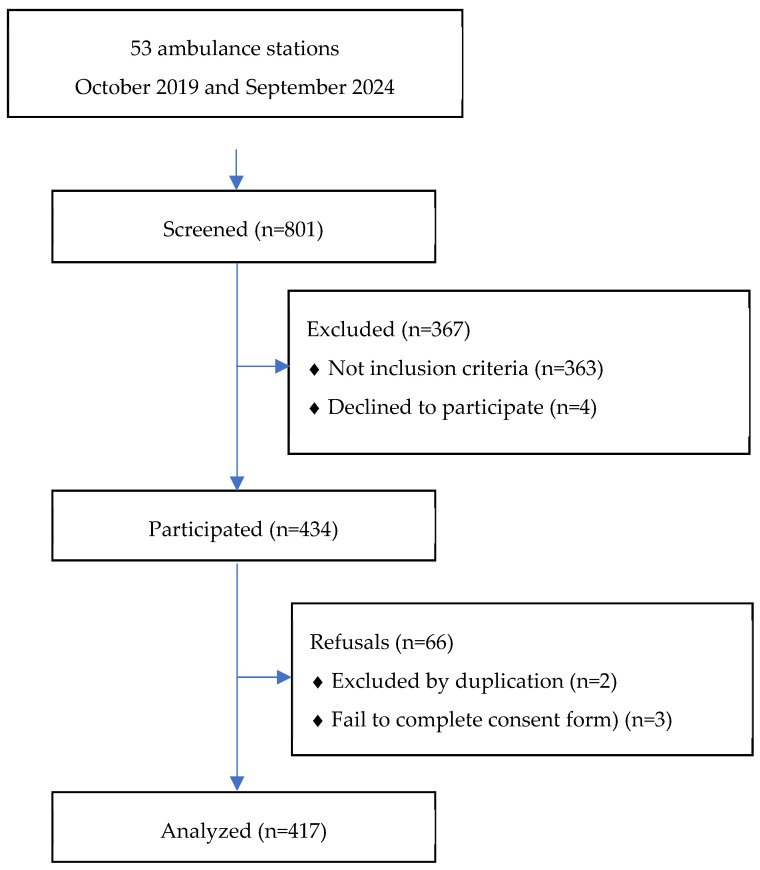
Study participation flowchart.

A total of 417 patients met the inclusion criteria. The patient selection process is detailed in Figure 1. The median age was 84 years (IQR 78–90), and 201 patients (48.2%) were female. Overall M2Y was 57.6%, increasing to 74.4% among patients with COPD. The clinical and epidemiological characteristics of the study population, as well as the differences between survivors and non-survivors, are presented in Table 1.

Surviving patients were referred from primary care more often than non-survivors. Patients residing in nursing homes had a significantly higher mortality rate. Non-survivors had significantly lower values of SpO_2_, SBP, DBP, and received a higher number of medications. HR was significantly higher among non-survivors. Survivors exhibited lower levels of potassium, glucose, NT-proBNP, C-reactive protein (CRP), creatinine, lactate, urea, and pH. Patients who died had a significantly higher age-adjusted aCCI and more frequently required non-invasive mechanical ventilation (NIMV) and invasive mechanical ventilation (IMV). Additionally, non-survivors were more frequently admitted to both hospital wards and ICUs. Of the total cohort, 417 patients (92.3%) had an elevated aCCI.

### 3.2. Survival Analysis

**Figure 2:**
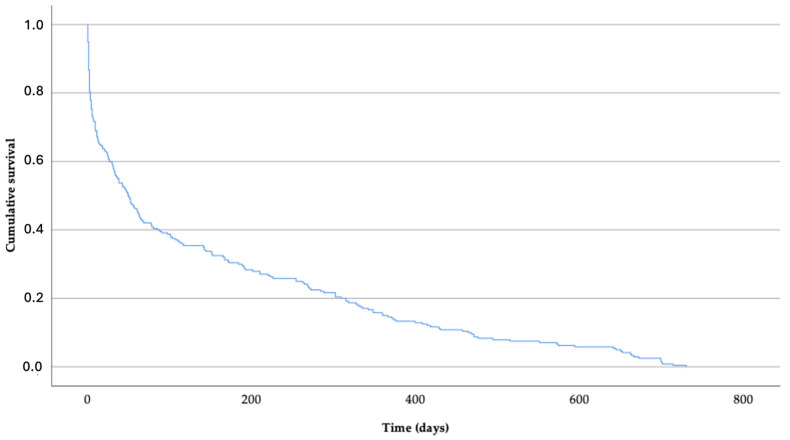
Kaplan–Meier survival curve—2-year mortality in the entire cohort.

The highest proportion of deaths occurred during the early stages of follow-up, particularly within the first two months. A total of 13.3% of patients died on the first day, 28.3% during the first week, and 50% within the first 49 days. The last recorded death occurred 714 days after the initial medical evaluation. Survival analysis for the overall cohort, performed using the Kaplan–Meier method, is presented in Table 2 and Figure 2.

**Figure 3:**
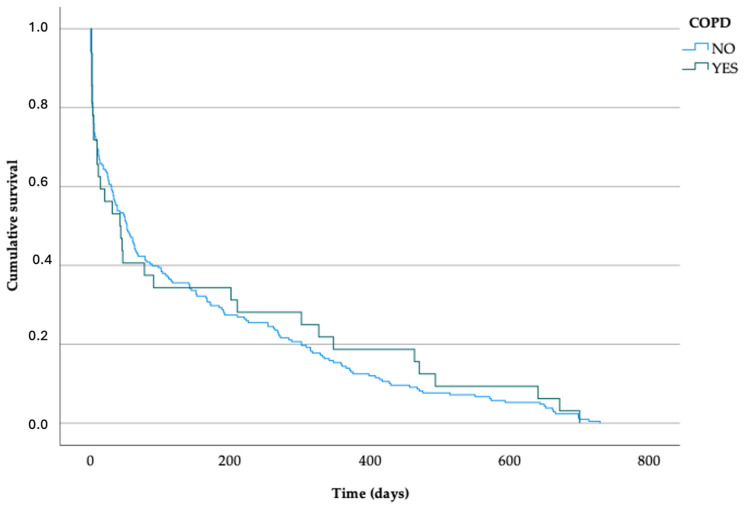
Survival function for COPD and not-COPD patients. Kaplan–Meier curves.

A survival analysis stratified by the presence or absence of COPD was also performed using the Kaplan–Meier method (Table 3 and Figure 3). The survival curves for both groups showed a similar distribution throughout the follow-up period, except on the first day, when 30 deaths (14.4%) occurred in the non-COPD group and 2 deaths (6.2%) in the COPD group.

A Cox regression analysis was performed for the variables under study, as shown in Table 4. In the resulting model, the variable most strongly associated with 2-year mortality was the aCCI range, with a hazard ratio of 2.865. Plasma potassium and lactate levels were also significantly associated with patient survival, although with lower hazard ratios.

## 4. Discussion

This prospective, multicenter, observational study is the first to analyze two-year survival in patients with acute heart failure (AHF) managed by EMSs. The main finding was the high mortality observed throughout the follow-up period, reaching 57.6% at two years. Li et al. [8] conducted a survival analysis of patients with AHF treated in EDs in Beijing, reporting a five-year mortality rate of 55.4%. Although comparable to our figure, this rate was observed at the end of a much longer follow-up period, and therefore, a substantially lower mortality at two years would be expected. In the study by Llorens et al. [9], which examined patients with AHF treated in hospital EDs in Spain, mortality was markedly lower—9.4% at 30 days and 29.5% at one year—compared to 23.7% and 48.9%, respectively, in our cohort. A more comparable analysis is that of Miró et al. [5], also conducted in Spanish hospital EDs, which included patients transported by advanced life support units. In their study, 30-day mortality was 15%, versus 23.7% in our cohort. Although both studies share certain similarities, differences in mortality may be explained by distinct characteristics of the included populations. In our study, patients were older (median age 84 years vs. mean age 80 x in Miró et al.) and had a significantly higher comorbidity burden, with 92.3% falling within the high-risk category of the aCCI. While functional status was not collected in our cohort, Miró et al. reported that 24.1% of patients had a Barthel Index < 60, which suggests a high level of dependency; our population may have had even greater baseline frailty. Another contributing factor may be the geographic setting: our study was conducted in Castilla y León, a region with one of the oldest populations in Spain [10], which likely influences both baseline frailty and medium- to long-term prognosis.

Another relevant finding of our study was the high two-year mortality observed among patients with COPD, which reached 74.4%. This is expected, as previous evidence has shown that patients with AHF and hypercapnia may experience higher mortality rates [11,12]. Nevertheless, the presence of COPD did not show a significant impact on survival in the Cox regression model and was therefore excluded during the univariate analysis stage. This is consistent with Jacob et al. [13], who also found no statistically significant association between COPD and mortality in ED-managed AHF patients, despite slightly higher crude mortality in COPD groups. However, the relatively small number of COPD patients in our cohort (n = 43) likely reduced statistical power, and the possibility of type II error must be acknowledged. COPD in this population may therefore reflect frailty and multimorbidity rather than acting as an independent determinant of survival. Furthermore, the high early mortality observed in both COPD and non-COPD patients (43.8% vs. 40.9% at 30 days) suggests that acute severity and underlying vulnerability may overshadow the relative contribution of COPD.

The survival analysis of the overall patient cohort also revealed a high concentration of deaths during the early days of follow-up. Specifically, 13.3% of patients died on the first day after prehospital care, 28.3% within the first week, and 50% before 49 days had elapsed. In the study by Li et al. [8] 22.6% of patients died within the first month. This temporal distribution suggests a critical period of heightened clinical vulnerability in the immediate aftermath of decompensation, which could benefit from targeted interventions aimed at early stabilization and a more structured transition of care between prehospital and hospital settings. Although no previous long-term survival analyses have been conducted in patients with acute heart failure (AHF) managed in the prehospital setting, the available data from other contexts report lower mortality. In the same study by, Li et al. [8] it was found that 22.6% of deaths occurred within the first month and 39.2% within the first year—figures considerably lower than those observed in our cohort (44.2% and 60.5%, respectively). These results underscore the unique nature of the prehospital setting and highlight the need for specific care approaches tailored to this patient profile, particularly during the earliest stages following initial contact with emergency medical services.

In our multivariate Cox regression model, the variables independently associated with patient survival were potassium, lactate, and, most notably, the aCCI, with hazard ratios of 1.45, 1.16, and 2.865, respectively. Lactate is a biomarker that has independently predicted mortality in patients treated in the prehospital setting for various conditions [14] as well as in those with acute cardiovascular disease [15]. It has therefore been incorporated into several prognostic scores for AHF to predict poor outcomes [16]. Potassium imbalances—both hypo- and hyperkalemia—are common in AHF, particularly in patients receiving diuretics or renin–angiotensin–aldosterone system inhibitors. Several studies have demonstrated that both low and high potassium levels are independently associated with increased risk of mortality and adverse outcomes in the short and long term, often following a U-shaped relationship, with the lowest risk observed in the range of 3.5–4.0 mmol/L [17,18,19]. These alterations may reflect not only a greater arrhythmic risk, but also renal dysfunction and overall clinical severity, all of which contribute to worse prognosis. Additionally, the aCCI quantifies overall comorbidity burden and the impact of aging on baseline patient status. A higher aCCI is associated with increased clinical frailty and reduced physiological reserve, limiting the capacity to respond to acute decompensation and leading to a higher risk of mortality and rehospitalization in patients with heart failure [20]. In the study by Li et al. [8] a multivariate analysis was also performed to identify independent predictors of mortality. Although potassium and lactate were not included in the final model, ischemic heart disease, cardiomyopathy, diabetes, and stroke—conditions encompassed by the aCCI—were identified as independent risk factors.

This study has several limitations that must be acknowledged. First, although the calculated sample size was 594 patients, only 417 were ultimately included. This shortfall reduces the statistical power of this study and may increase the risk of type II error, particularly in subgroup analyses such as COPD. Second, although inclusion was continuous, this study relied on convenience sampling, which introduces potential selection bias. Third, this study was conducted in a single Spanish region (Castilla y León), characterized by one of the oldest populations in Europe, which limits external validity and generalizability to younger AHF populations or to regions with different demographic profiles. Fourth, while survival follow-up was complete, several important clinical variables were not collected, including functional status, heart failure phenotype, precipitating factors of AHF, in-hospital management, rehospitalizations, and cause of death. The absence of these variables limits the depth of interpretability regarding mechanisms of mortality and may mask potential confounding effects. Fifth, the markedly advanced age of the cohort means that advanced mechanical circulatory support (e.g., extracorporeal membrane oxygenation (ECMO), Impella) is generally not indicated, and therefore our results cannot be extrapolated to younger AHF patients or those eligible for invasive therapies. Finally, due to the high early mortality rate, acute severity may have overshadowed subtler chronic factors, limiting the identification of additional prognostic variables.

## 5. Conclusions

Patients with AHF attended by EMSs show a notably high 2-year mortality, reaching 57.6%, and rising to 74.4% among those with COPD. Half of the deaths occurred within the first 49 days, highlighting a critical early phase of vulnerability. Elevated plasma potassium and lactate levels, along with a higher aCCI, were independently associated with reduced survival. These findings suggest that comorbidity burden and early metabolic derangements are key determinants of poor outcomes in prehospital AHF patients and should be considered when developing risk stratification tools and early intervention strategies.

## Figures and Tables

**Table 1:** Baseline patient’s characteristics based on 2-year mortality.

	2-Year Mortality			
No. with Data ^a^	Total 417	Survivors 177 (42.4)	Non-Survivors 240 (57.6)	*p* Value ^b^
Sex, Female (%)	201 (48.2)	97 (48.3)	104 (51.7)	0.021
Age, year	84 (78–90)	79 (72–85)	80 (71–88)	<0.001
Primary health care (%)	195 (25.2)	54 (30.5)	51 (21.3)	0.031
Nursing homes (%)	111 (26.6)	38 (21.5)	73 (30.4)	0.041
Rural zone (%)	109 (26.1)	53 (29.9)	56 (23.3)	0.129
COPD (%)	43 (10.3)	11 (25.6)	32 (74.4)	0.018
Baseline vital signs				
RR (breathings/min)	29.5 (17–35.7)	32 (21–40)	29 (24–36)	0.053
SpO_2_ (%)	93 (77–99)	92 (84–95)	85 (75–94)	<0.007
FiO_2_ (%)	0.21 (0.21–0.81)	0.21 (0.21–0.5)	0.21 (0.21–0.31)	<0.001
SBP (mmHg)	140.5 (101.5–195.5)	162 (128–207)	136 (106–178)	<0.001
DBP (mmHg)	69.5 (54.7–101)	93 (73–109)	75 (60–94)	<0.001
HR (beats/min)	63 (51.5–82.2)	100 (80–129)	104 (76–126)	0.013
Temperature (°C)	35.9 (35–36–5)	36.1 (35.7–36.7)	36.1 (35.7–36.7)	0.327
GCS (puntos)	15 (14–15)	15 (15–15)	15 (14–15)	<0.001
Drugs (N°)	3 (2–6)	4 (2–6)	3 (4–5)	<0.001
Prehospital blood analysis				
Sodium	140 (136.2–141.7)	138 (135–141)	138 (134–141)	0.8
Potassium	4.8 (4.4–5.7)	4.2 (4–4.5)	4.4 (3.9–5.1)	<0.001
Glucose	152 (130.7–226.25)	155 (130–199)	168 (134–255)	0.002
D-dimer	3903.5 (915.2–22,822)	1182 (733–1801)	1210 (740–3937)	0.237
NT-proBNP	11,918.5 (3489.5–28,975.7)	3792 (2076–5712)	6169 (3167–16,207)	<0.001
cTn	111.6 (33.2–186.8)	49.5 (14.1–215.2)	69.4 (32.8–210.8)	0.791
PCR	18.5 (9.6–43.3)	24.3 (7–75.5)	62.35 (20.26–133)	<0.001
Creatinine (mg/dL)	1.38 (1.08–1.69)	1.22 (0.89–1.71)	1.45 (1.02–2.34)	<0.001
Lactate (mmol/L)	2.65 (1.27–4.24)	1.6 (1.4–2.3)	2.3 (1.7–5.2)	<0.001
Urea (mg/dL)	84.1 (68.1–99.3)	67.8 (48–81.1)	76.4 (45.7–98)	<0.001
pH	7.2 (7.2–7.3)	7.38 (7.32–7.48)	7.34 (7.23–7.4)	<0.001
pCO_2_	44.5 (43–60.5)	42 (836–49)	46.3 (38–58.75)	0.10
pO_2_	74 (23.2–174)	65.5 (53.25–98.75)	63 (38.5–89)	0.19
HCO_3_^−^	2.6 (17–29.1)	24 (22.12–26.07)	23.45 (20–27.95)	0.270
aCCI (points)	7 (6–9)	5 (3–7)	8 (6–10)	<0.001
aCCI range (points)				
Low (1–2)	4 (1)	3 (1.7)	1 (0.4)	0.002
Medium (3–4)	28 (6.7)	20 (11.3)	8 (3.3)	
High (≥5)	417 (92.3)	154 (87)	231 (96.3)	
Pre-hospital oxygen therapy support (%) ^c^				
Nasal-cannula	54 (12.9)	26 (14.7)	28 (11.7)	0.364
Venturi mask	96 (23)	45 (25.4)	51 (21.3)	0.317
Nonrebreather mask	14 (3.4)	7 (4)	7 (2.9)	0.561
NIMV	138 (33.1)	39 (22)	99 (41.3)	0.002
IMV	20 (4.8)	4 (1)	16 (6.7)	0.037
Inpatient (%)	369 (88.5)	146 (82.5)	223 (92.9)	<0.001
ICU admission (%)	94 (22.5)	40 (42.6)	54 (57.4)	<0.0981

RR: respiratory rate; SpO_2_: pulse oximetry saturation FiO_2_: fraction of inspired oxygen; SBP: systolic blood pressure; DBP: diastolic blood pressure; HR: heart rate; GCS: Glasgow Coma Scale; cTn: Cardiac troponin NT-proBNP: N-terminal portion of B-type natriuretic peptide; aCCI: Age–adjusted Charlson comorbidity index; NIMV: noninvasive mechanical ventilation; IMV: invasive mechanical ventilation; ICU: intensive care unit. ^a^ Values are expressed as the total number (percentage) and median (25th percentile–75th percentile), as appropriate. ^b^ The Mann—Whitney U test or chi-squared test was used as appropriate. ^c^ Multiple oxygen therapy systems could be used for a single patient.

**Table 2:** Survival analysis—2-year mortality in the entire cohort. Kaplan–Meier method.

Time (Days)	Cumulative Survival (%) ^a^	Deaths (%) ^b^	Mortality (%)
1	86.7	32 (13.3)	7.7
2	80.4	47 (19.6)	11.3
7	71.7	68 (28.3)	16.3
30	58.8	99 (44.2)	23.7
49	50	120 (50)	28.9
90	39.2	146 (60.8)	35
180	30.4	167 (69.6)	40
365	15	204 (85)	48.9
714	0	240 (100)	57.6

^a^ Percentage of survivors in relation to deceased patients. ^b^ Percentage of deaths in relation to the total number of patients.

**Table 3:** Survival analysis for 2-year mortality in COPD and not-COPD patients. Kaplan–Meier method.

Time (Days)	Cumulative Survival (%) No COPD ^a^	Deaths (%) No COPD ^b^	Mortality (%) No COPD	Cumulative Survival (%) COPD ^a^	Deaths (%) COPD ^b^	Mortality (%) COPD
1	85.6	30 (14.4%)	8	93.8	2 (6.2%)	4.7
2	80.3	41 (19.7%)	11	81.3	6 (18.8%)	14
7	71.6	59 (28.4%)	15.8	71.9	9 (28.1%)	20.9
30	59.1	85 (40.9%)	22.7	56.3	14 (43.8%)	32.6
90	39.9	125 (60.1%)	33.4	34.4	21 (65.6%)	48.8
180	29.8	146 (70.2%)	39	34.4	21 (65.6%)	48.8
365	14.4	178 (85.6%)	47.6	18.8	26 (81.2%)	60.5
730	0	208 (100.0%)	64.2	0	32 (100.0%)	74.4

^a^ Percentage of survivors in relation to deceased patients. ^b^ Percentage of deaths in relation to the total number of patients.

**Table 4:** Cox regression for the variables in overall patients. ^a^ Wald test.

Variable	Wald ^a^	Hazard Ratio	*p* Value
Age	0.43	1.01	0.46
SpO_2_	3.05	1.02	0.08
SBP	1.01	0.99	0.31
DBP	0.04	0.99	0.82
GCS	0.02	0.99	0.87
Potassium	5.32	1.45	0.02
Creatinine	0.94	0.85	0.33
Tn	1.66	1	0.19
CRP	0.03	1	0.87
Urea	0.36	0.99	0.54
Lactate	12.97	1.16	<0.001
aCCI range	3.853	2.865	0.05

SpO_2_: pulse oximetry saturation; SBP: systolic blood pressure; DBP: diastolic blood pressure; GCS: Glasgow coma scale; Tn: Troponins; CRP: C reactive protein; aCCI range aCCI: Age–adjusted Charlson comorbidity index.

## Data Availability

Details of the study design, statistical analysis plan, and underlying. Raw data can be made available upon reasonable request.

## Supplementary Materials

The following supporting information can be downloaded at: https://www.mdpi.com/article/10.3390/jcdd12120485/s1, Table S1: STROBE statements, Table S2: Age-adjusted Charlson Comorbidity Index calculation.

## Abbreviations

The following abbreviations are used in this manuscript:

aCCI	Age-adjusted Charlson comorbidity index
AECOPD	Acute exacerbation of chronic pulmonary obstructive disease
AHF	Acute heart failure
BLS	Basic life support
BUN	Blood urea nitrogen
CI	Confidence interval
COPD	Chronic obstructive pulmonary disease
DBP	Diastolic blood pressure
ED	Emergency department
EMTs	Emergency medical technicians
EMSs	Emergency medical services
ERN	Emergency registered nurse
FiO_2_	Fraction of inspired Oxygen
GCS	Glasgow coma Scale
HF	Heart failure
HR	Heart rate
ICU	Intensive care unit
IMV	Invasive mechanical ventilation
IQRs	Interquartile ranges
M1Y	One-year mortality
M2Y	Two-year mortality
NEWS2	National Early Waring Score 2
NIMV	Noninvasive mechanical ventilation
RR	Respiratory rate
SACYL	Castilla y León Public Health System
SBP	Systolic blood pressure
SpO_2_	Peripheral oxygen saturation
STROBE	STrengthening the Reporting of OBservational studies in Epidemiology
TT	Temperature
